# MLFLHMDA: predicting human microbe-disease association based on multi-view latent feature learning

**DOI:** 10.3389/fmicb.2024.1353278

**Published:** 2024-02-02

**Authors:** Ziwei Chen, Liangzhe Zhang, Jingyi Li, Mingyang Fu

**Affiliations:** School of Electronic and Information Engineering, Beijing Jiaotong University, Beijing, China

**Keywords:** microbe, disease, microbe-disease association, multi-view, latent feature learning

## Abstract

**Introduction:**

A growing body of research indicates that microorganisms play a crucial role in human health. Imbalances in microbial communities are closely linked to human diseases, and identifying potential relationships between microbes and diseases can help elucidate the pathogenesis of diseases. However, traditional methods based on biological or clinical experiments are costly, so the use of computational models to predict potential microbe-disease associations is of great importance.

**Methods:**

In this paper, we present a novel computational model called MLFLHMDA, which is based on a Multi-View Latent Feature Learning approach to predict Human potential Microbe-Disease Associations. Specifically, we compute Gaussian interaction profile kernel similarity between diseases and microbes based on the known microbe-disease associations from the Human Microbe-Disease Association Database and perform a preprocessing step on the resulting microbe-disease association matrix, namely, weighting K nearest known neighbors (WKNKN) to reduce the sparsity of the microbe-disease association matrix. To obtain unobserved associations in the microbe and disease views, we extract different latent features based on the geometrical structure of microbes and diseases, and project multi-modal latent features into a common subspace. Next, we introduce graph regularization to preserve the local manifold structure of Gaussian interaction profile kernel similarity and add $L_{p,q}$ -norms to the projection matrix to ensure the interpretability and sparsity of the model.

**Results:**

The AUC values for global leave-one-out cross-validation and 5-fold cross validation implemented by MLFLHMDA are 0.9165 and 0.8942+/−0.0041, respectively, which perform better than other existing methods. In addition, case studies of different diseases have demonstrated the superiority of the predictive power of MLFLHMDA. The source code of our model and the data are available on https://github.com/LiangzheZhang/MLFLHMDA_master.

## Introduction

The interactions between biological activities and complex, diverse and dynamically changing microbial communities are intricate (Sommer and Backhed, 2013). On one hand, the relationship between humans and the microbiome is mutualistic, for example, gut microbes can synthesize essential amino acids and vitamins required by the human body and also facilitate the digestion and absorption of less easily digestible foods (Huang et al., 2017). Furthermore, there is clinical and histological evidence suggesting that the topical application of lactic acid can effectively depigment, improve skin surface roughness, and reduce mild wrinkles caused by environmental photodamage (Huang et al., 2020). On the other hand, compelling evidence suggests that disruptions in the host microbial community can increase the incidence of various complex human diseases, such as diabetes (Wen et al., 2008), asthma (Noval Rivas et al., 2016), liver diseases (Henao-Mejia et al., 2013), and even cancers (Castellarin et al., 2012; Schwabe and Jobin, 2013). Some researchers have found that dysbiosis is associated with overgrowth of microbes such as *S. aureus*, which employs clumping factor B (ClfB), toxins, proteases, and superantigens to colonize the skin and induce damaging inflammatory responses (Edslev et al., 2020). In addition, other studies have shown that Clostridia responds to various physiological signals and secrete Large Clostridial Toxins (LCTs), which are considered the major virulence factors for various infections.

As mentioned above, identifying the potential relationship between microbes and diseases will be beneficial in elucidating the pathogenesis of diseases and providing new medical solutions for disease prevention, diagnosis, and treatment. However, traditional approaches often require a significant expenditure of cost and time to establish novel associations between microbes and diseases through biological or clinical experiments (Chen et al., 2019). With the advancement of computer technology, it has become imperative to predict potential microbe-disease associations by constructing computational models. The HMDAD database, established by Ma et al. (2017), through manual curation from large-scale public literature, is the first human microbe-disease association database. Based on this database, excellent computational models can be developed to prioritize potential microbes for large-scale research on disease associations. Researchers have successively proposed microbe-disease prediction models based on different theories, which could be broadly categorized into the following three types (Zhao et al., 2021): (1) score function-based models, (2) network algorithm-based models, and (3) machine learning-based models.

In the score function-based models, the probability of association between diseases and microbes is calculated using score functions based on various methods. Chen et al. (2017) built a microbe-disease association network and proposed a novel computational model of the KATZ measure for Human Microbe-Disease Association prediction (KATZHMDA). In this model, the prediction of latent associations was transformed into calculating the similarity between corresponding nodes based on the lengths and quantities of paths connecting two nodes in the network. Huang et al. (2017) developed a novel computational model based on a depth-first search algorithm for predicting microbes potentially associated with diseases (PBHMDA). The authors initially established a heterogeneous network by integrating known microbe-disease associations and Gaussian interaction profile kernel similarities between microbes and diseases. Then, a specialized depth-first search algorithm was employed to traverse all connected paths between nodes in the heterogeneous network, thereby obtaining prediction scores for each microbe-disease association pair. Long and Luo (2019) developed a novel computational model for predicting disease-associated microbes (WMGHMDA) based on weighted meta-graph. In the model, the authors defined the contribution value of the association probability for a given microbe-disease pair as the product of weights of all edges included in the meta-graph. Subsequently, the sum of contribution values from all meta-graphs for a given microorganism-disease pair was used as its final prediction score. The advantages of these models are that the theory of the algorithms and computational processes involved are relatively easy to understand, and the models do not require negative samples for prediction, which is extremely difficult to obtain.

The second type of method is network algorithm-based models, Bao et al. (2017) proposed a computational method named NCPHMDA to infer latent microbes for diseases by calculating consistency projection scores. The model measured the correlation between microbes and diseases by calculating the similarity of nodes in the heterogeneous network. Wu et al. (2018) introduced a novel model for optimizing random walks and restarts on the human microbe-disease association heterogeneous network (PRWHMAD). The heterogeneous network consisted of disease networks and microbe networks from different data sources, respectively. Finally, the authors used particle swarm optimization (PSO) to optimize the parameters of the random walk and obtain the final vector of association probabilities. Niu et al. (2019) proposed the Random Walk on Hypergraph for Microbe-Disease Association (RWHMDA) model to predict potential microbe-disease associations. Specifically, Niu and colleagues constructed a novel higher-order hypergraph model and extended the well-known random walk process to hypergraphs in a modified manner. Yan et al. (2020) introduced BRWMDA, a correlation prediction method based on the similarity between microbes and diseases. The approach utilized network integration and dual random walks on disease and microbe networks. The random walks ceased when the maximum number of iterations for both networks was reached, yielding the final correlation probability matrix. Wang Y. et al. (2022) presented the MSLINE model, which constructed a Microbe Disease Heterogeneous Network (MDHN) by integrating known associations and multiple similarities. Subsequently, a random walk algorithm was implemented on the MDHN to learn its structural information. Finally, the microbe-disease associations were scored based on the structural information of each node. The main advantage of these models is that they can fully utilize the topological information in the network. In addition, these models involve fewer parameters, which greatly reduces the difficulty of parameter selection.

The third kind of approach is based on machine learning. In recent years, machine learning has been applied in bioinformatics and computational biology, such as in miRNA-disease association prediction (Liu et al., 2022; Wang C.-C. et al., 2022), metabolite-disease association prediction (Sun et al., 2022; Gao et al., 2023), miRNA-lncRNA association prediction (Wang W. et al., 2022), and lncRNA-protein association prediction (Zhao et al., 2023). To some extent, these studies have contributed to the development of computational models for predicting microbe-disease associations. For example, Peng et al. (2018) proposed a novel model called ABHMDA, which revealed microbes associated with diseases through a strong classifier composed of weak classifiers with respective weights. ABHMDA assigned different weights to multiple weak classifiers and obtained the final association. Wang et al. (2017) introduced a LRLSHMDA calculation method based on machine learning, calculated the association probability of microbe-disease pairs based on the observed microbe-disease relationship network, and used it to prioritize all candidate microbes for the diseases studied, and achieved good results. Li et al. (2021) proposed a novel calculative method called BPNNHMDA, which utilized a neural network model with a unique activation function and optimized initial connection weights based on Gaussian interaction profile kernel similarity to predict potential microbe-disease associations.

While researchers continue to explore potential microbe-disease relationships, current computational models still have some limitations. Firstly, most scoring function-based models are not applicable to new diseases. Some network-based methods heavily rely on experimentally validated microbe-disease associations and are unable to predict new diseases or microbes in the absence of known association information. Secondly, the microbe-disease association network is often sparse, which hinders the prediction of microbe-disease associations (MDAs). Additionally, many methods struggle to effectively extract feature matrices from the microbe-disease association matrix, resulting in a diminished model generalization ability. Lastly, some methods focus solely on a single representation of the disease or microbe space, which can negatively impact the predictive performance of the model.

**Figure 1:**
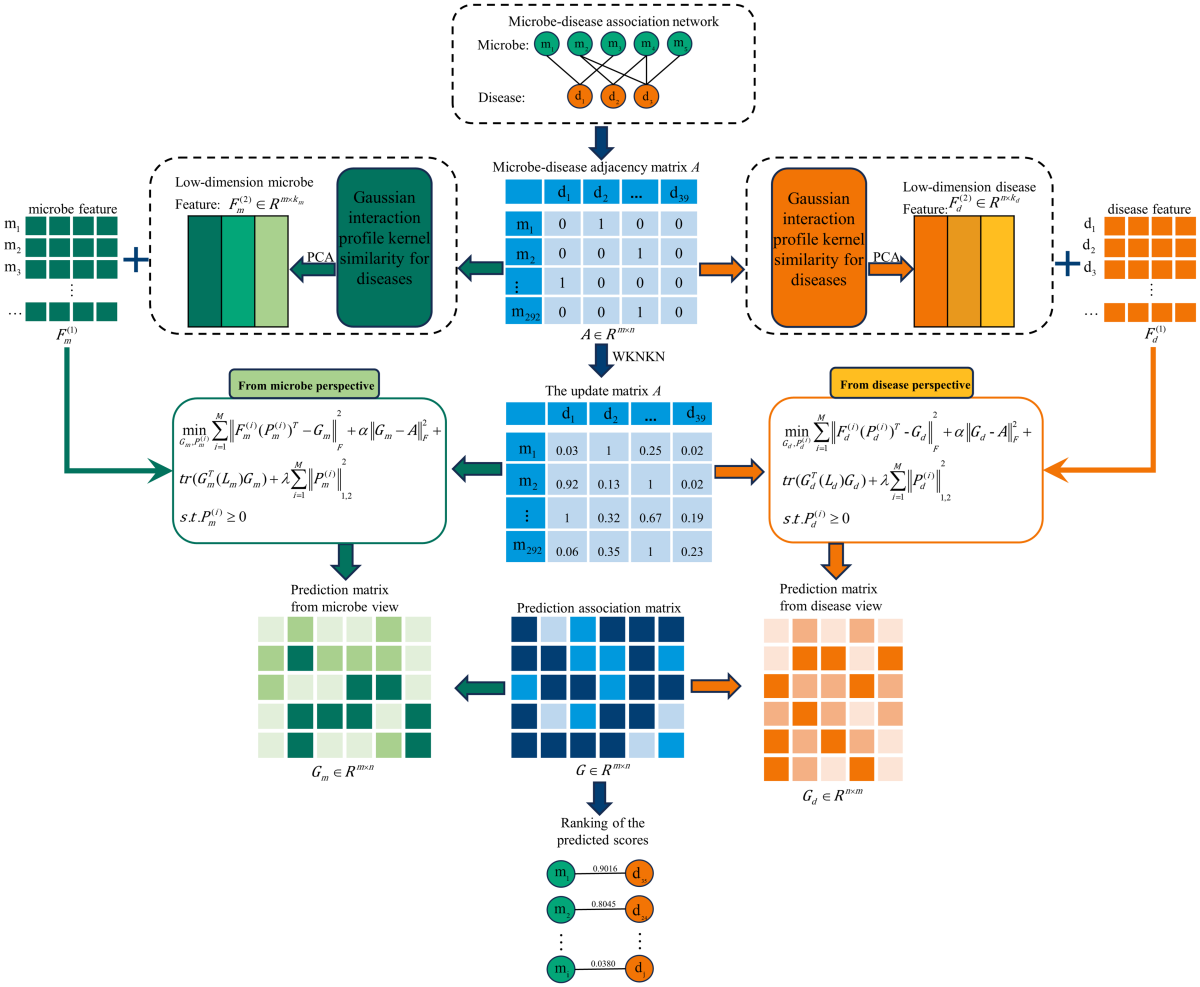
Flowchart of potential microbe-disease association prediction based on the computational model of MLFLHMDA.

In this study, we introduce a novel approach using Multi-View Latent Feature Learning for Human Microbe-Disease Association prediction (MLFLHMDA) to reveal the associations between diseases and microbes. The approach takes a disease and microbe view to infer the microbes associated with the disease. Figure 1 shows the flowchart of the MLFLHMDA. Firstly, the method constructs the microbe-disease association matrix and Gaussian interaction profile (GIP) kernel similarity from known MDAs. Considering that the sparsity of MDAs is not conducive to ensemble learning, the weighted K nearest known neighbors (WKNKN) method is used to preprocess the microbe-disease association matrix. Secondly, we employ Principal Component Analysis (PCA) on GIP kernel similarity of microbes and diseases to extract potential features of microbes and diseases. We then project multi-modal latent features into a common subspace of the microbe and disease spaces and utilize an integrated latent feature learning approach to infer potentially disease-associated microbes. Furthermore, we enhance model interpretability and performance by incorporating graph regularization and implementing $L_{p,q}$ -norm constraints in the learning task. Finally, to optimize the learning problem, we use an alternate iteration algorithm and score and rank each microbe and disease pair. In the global leave-one-out cross-validation (LOOCV) and 5-fold cross validation, MLFLHMDA has a good performance with the area under the receiver operating characteristic curve (AUC) of 0.9165 and 0.8942+/−0.0041, respectively. In addition, case studies of four different diseases further demonstrate that the MLFLHMDA is a useful tool to effectively identify potential MDAs.

The main contributions of this paper are as follows:

- Firstly, we provide a new approach to multi-view latent feature learning that infers disease-associated microbes from a microbe and disease view and combines similarity features, multi-modal latent features, and known association information.
- Secondly, we perform graph regularization on the similarity features to efficiently capture the graph structure information in the data space and to constrain the learning task.
- Finally, to improve the interpretability of the model and to mitigate the effect of noise inherent in the feature space of microbes and diseases, we impose $L_{p,q}$ -norms on the projection matrix to obtain the most representative sparse features.

## Materials and methods

### Human microbe-disease associations

We download the database from HMDAD^1^ (Ma et al., 2017), which contains 483 confirmed associations involving 292 microbes and 39 diseases. However, the dataset has some duplicate associations. After removing duplicate records, we ultimately obtain 450 unique associations. Next, we construct the adjacency matrix $A\in R^{m\times n}$ for the microbe-disease association network, with the variables m and n representing the quantities of microbes and diseases under study, respectively. A(i,j) is equal to 1 if there is a known association between microbe m(i) and disease d(j) , otherwise, the value is 0. In the adjacency matrix A , each row binary vector $A\left(m_{i}\right)$ corresponds to a microbe, and each column vector $A\left(d_{j}\right)$ corresponds to a disease.

### Gaussian interaction profile kernel similarity

We operate under the assumption that diseases with similar characteristics are likely to have associations with functionally similar microbes and that there are analogous patterns of interaction and non-interaction between diseases and microbes (Chen and Yan, 2013). To capture the microbe similarity, we construct the Gaussian interaction profile kernel similarity for microbes, denoted as SM . Let $IP\left(m_{i}\right)$ and $IP\left(m_{j}\right)$ represent the binary interaction profile vectors of microbes m(i) and m(j) , corresponding to the i - th and j - th rows in the adjacency matrix A . The Gaussian interaction profile kernel similarity between microbe m(i) and m(j) is computed based on their interaction profiles as shown in Eq. (1).

(1) $$SM\left(m\left(i\right),m\left(j\right)\right)=\exp\left(-\gamma_{m}||IP\left(m\left(i\right)\right)-IP\left(m\left(j\right)\right)|{|}^{2}\right)$$

here parameter $\gamma_{m}$ is used to regulate the kernel bandwidth, and it introduces another parameter $\gamma_{m}^{\prime }$ , to denote the average quantity of all microorganisms related to diseases. The calculation of $\gamma_{m}$ is calculated as follows:

(2) $$\gamma_{m}=\frac{\gamma_{m}^{\prime }}{\frac{\sum_{i=1}^{m}||IP\left(m\left(i\right)\right)|{|}^{2}}{m}}$$

where the value of $\gamma_{m}^{\prime }$ is set to 1 in Eq. (2). The definition of GIP kernel similarity for disease SD is similar to SM .

### Network-based multi-modal feature extraction

Based on known microbe-disease associations, we construct the adjacency matrix A and similarity networks to facilitate the extraction of multi-modal features for microbes and diseases. The adjacency matrix A reflects the associations between each microbe. The GIP kernel similarity of microbes (or diseases) not only contains similar information but also valuable information for the construction of latent features. Therefore, we use it to extract an alternative set of latent features for both microbes and diseases. Furthermore, given that as the dimensionality increases, data sparsity becomes more pronounced, Principal Component Analysis (PCA) can effectively project the data into a lower-dimensional subspace to reduce dimensionality by finding the most dominant direction of variance in the data (Lu et al., 2018). Therefore, we perform additional latent extraction using PCA on $SM\in R^{m\times m}$ and $SD\in R^{n\times n}$ and utilize singular value decomposition (SVD) to decompose PCA. Since SM and SD are symmetric, they can be decomposed as $USU^{T}$ , where U is a unitary matrix and S is a diagonal matrix with singular values arranged in descending order along the diagonal. Following the dominating energy strategy, we extract $g_{m}$ and $g_{d}$ as the dimensions, denoting them as $F_{m}\left(m_{i}\right)=\left\{f_{i1},f_{i2},\ldots ,f_{ig_{m}}\right\}$ and $F_{d}\left(d_{i}\right)=\left\{f_{1i},f_{2i},\ldots f_{g_{d}i}\right\}$ , which represent the low-dimensional feature vectors for microbe $m_{i}$ and disease $d_{i}$ . The dimensions $g_{m}$ and $g_{d}$ are defined as shown in Eqs. (3) and (4):

(3) $$g_{m}=\begin{array}{c} \arg\;\min\\ x \end{array}\left(\frac{\sum_{i=1}^{x}{\left(SM\right)}_{ii}}{\sum_{j=1}^{m}{\left(SM\right)}_{jj}}\geq \sigma_{m}\right)$$

and

(4) $$g_{d}=\begin{array}{c} \arg\;\min\\ x \end{array}\left(\frac{\sum_{i=1}^{x}{\left(SD\right)}_{ii}}{\sum_{j=1}^{m}{\left(SD\right)}_{jj}}\geq \sigma_{d}\right)$$

here $\sigma_{m}$ and $\sigma_{d}$ are set to 0.7 as suggested by Xiao et al. (2020).

### MLFLHMDA

Inspired by this article Xiao et al. (2020), the main idea of MLFLHMDA is to integrate the views of microbes and diseases by using similarity features and multi-modal latent features in learning for disease-related microbe inference tasks. The main symbol descriptions are listed in Table 1.

**Table 1:** Symbol description.

Notation	Definition
m	Number of microbes
n	Number of diseases
$A\in R^{m\times n}$	Known microbe-disease association matrix
$SM\in R^{m\times m}$	Microbe Gaussian interaction profile kernel similarity
$SD\in R^{n\times n}$	Disease Gaussian interaction profile kernel similarity
$g_{m}^{\left(i\right)}\left(org_{d}^{\left(i\right)}\right)$	Dimension of the i - th microbe (or disease) feature matrix
$F_{m}^{\left(i\right)}\in R^{m\times g_{m}^{\left(i\right)}}\left(or\;F_{d}^{\left(i\right)}\in R^{n\times g_{d}^{\left(i\right)}}\right)$	The i - th microbe (or disease) feature matrix
$P_{m}^{\left(i\right)}\in R^{n\times g_{m}^{\left(i\right)}}\left(or\;P_{d}^{\left(i\right)}\in R^{m\times g_{d}^{\left(i\right)}}\right)$	The projection matrix of the i - th microbe (or disease) feature
α,λ	Regularization parameters
$G_{m}\in R^{m\times n}\left(\mathrm{or}\;G_{d}\in R^{n\times m}\right)$	The common latent interaction matrix of microbe (or disease)
$G\in R^{m\times n}$	The predicted microbe-disease association matrix

Firstly, we integrate the GIP similarity matrices for microbes SM and diseases SD . Subsequently, we employ Principal Component Analysis to extract low-dimensional latent feature matrices from these matrices. Secondly, the available information on microbe-disease associations is highly limited. The resulting adjacency matrix A is sparse, and the values in the interaction profiles $IP\left(m_{i}\right)$ and $IP\left(d_{i}\right)$ for novel microbes or diseases are all zeros. Therefore, we employ a preprocessing step called Weighted K-Nearest Known Neighbors (WKNKN) to transform the microbe-disease associations matrix A into values ranging from 0 to 1 (Wu et al., 2020). Based on the functional similarity between microbe $m_{q}$ and its K nearest known neighbors, we obtain the interaction profile for each microbe $m_{q}$ as follows:

(5) $$A_{m}\left(m_{q},:\right)=\frac{1}{Q_{m}}\overset{K}{\underset{i=1}{\sum }}w_{i}^{\left(m\right)}A\left(m_{i}\right)$$

where $m_{1}$ to $m_{k}$ are the K nearest known neighbors arranged in descending order based on their similarity to $m_{q}$ ; $w_{i}^{m}$ represents the weight coefficient, and $w_{i}^{m}=\alpha^{i-1}*SM\left(m_{i},m_{q}\right)$ . α∈[0,1] denotes a decay term, and $Q_{m}=\overset{K}{\underset{i=1}{\sum }}SM\left(m_{i},m_{q}\right)$ is the normalization term. Similarly, the interaction profile for each disease $d_{q}$ is as follows:

(6) $$A_{d}\left(:,d_{q}\right)=\frac{1}{Q_{d}}\overset{K}{\underset{i=1}{\sum }}w_{i}^{\left(d\right)}A\left(d_{i}\right)$$

where $d_{1}$ to $d_{k}$ are the K nearest known neighbors arranged in descending order based on their similarity to $d_{q}$ ; $w_{i}^{d}$ is the weight coefficient, and $w_{i}^{d}=\alpha^{i-1}*SD\left(d_{i},d_{q}\right)$ . $Q_{d}=\overset{K}{\underset{i=1}{\sum }}SD\left(d_{i},d_{q}\right)$ is the normalization term. Following Xiao et al. (2018), K is set to 5 in the data space of microbes and diseases.

Then, in Eq. (7), we integrate $A_{m}$ and $A_{d}$ obtained separately from the two datasets mentioned above, replace $A_{ij}=0$ with an associated likelihood score, then the original adjacency matrix A can be updated by taking the average of the updated interaction likelihood profiles:

(7) $$A_{md}=\left(\varsigma_{1}A_{m}+\varsigma_{2}A_{d}\right)/\sum \varsigma_{i}\left(i=1,2\right)$$

(8) $$A_{ij}=\max\left(A_{ij,}\;{\left(A_{md}\right)}_{ij}\right)$$

where $\varsigma_{i}$ is the weight coefficient and $\varsigma_{1}$ = $\varsigma_{2}$ = 1.

### Mathematical formulation

In the microbe view, there are two types of feature matrices: $F_{m}^{1}$ represents the adjacency matrix A and $F_{m}^{2}$ represents the low-dimensional feature matrix obtained after dimension reduction. To consider the different features of microbes, we use a linear transformation to project these two distinct feature matrices into a common latent interaction subspace. By using $F_{m}^{\left(i\right)}{\left(P_{m}^{\left(i\right)}\right)}^{T}$ , where $P_{m}^{\left(i\right)}\in R^{n\times g_{m}^{\left(i\right)}}$ is the projection matrix of the i - th microbe feature matrix, i = 1,2. Thus, the common latent interaction matrix $G_{m}\in R^{m\times n}$ of microbe view can be approximately by $F_{m}^{\left(i\right)}{\left(P_{m}^{\left(i\right)}\right)}^{T}$ , which can be expressed as ${\left\|F_{m}^{\left(i\right)}{\left(P_{m}^{\left(i\right)}\right)}^{T}-G_{m}\right\|}_{F}^{2},i=1,2,$ where ${\left\|\cdot \right\|}_{F}$ represents the Frobenius norm.

Furthermore, the matrix $G_{m}$ is also designed to approximate the adjacency matrix A. Therefore, the following objective function can be represented mathematically as Eq. (9):

(9) $$\begin{array}{l} \underset{G_{m},P_{m}^{\left(i\right)}}{\min}\overset{M}{\underset{i=1}{\sum }}{\left\|F_{m}^{\left(i\right)}{\left(P_{m}^{\left(i\right)}\right)}^{T}-G_{m}\right\|}_{F}^{2}+\alpha {\left\|G_{m}-A\right\|}_{F}^{2}\;\\ s.t.P_{m}^{\left(i\right)}\geq 0 \end{array}$$

where α≥ 0 is a regularization parameter and M =2.

It has been shown that the graph regularization can effectively utilize the geometric structure of data to ensure a part-based representation (Gao et al., 2020). Here, we perform the graph regularization on the microbe similarity matrix to capture graph structural information (Cai et al., 2010). The graph regularization for microbe is defined as shown in Eq. (10):

(10) $$tr\left(G_{m}^{T}\left(D_{m}-SM\right)G_{m}\right)=tr\left(G_{m}^{T}L_{m}G_{m}\right)$$

(11) $$L_{m}=D_{m}-SM$$

where $L_{m}$ is the graph Laplacian matrix of SM in Eq. (11), and ${\left(D_{m}^{\left(v\right)}\right)}_{ii}=\overset{m}{\underset{j=1}{\sum }}{\left(SM\right)}_{ij}$ is a diagonal matrix.

Research has indicated that the $L_{p,q}$ mixed-norm can mitigate the influence of inherent noise in data space and filter out sparse features with high correlation which can have the effect of improving the interpretability of the model (Zhang et al., 2020). The projection matrix $P_{m}^{\left(i\right)}$ represents the weights of microbe features. Therefore, imposing the $L_{p,q}$ -norm on the projection matrix $P_{m}^{\left(i\right)},i=1,2,$ served to reduce its sparsity. The definition of $L_{p,q}$ -norm is as shown in Eq. (12):

(12) $${\left\|P_{m}^{\left(i\right)}\right\|}_{p,q}={\left(\overset{n}{\underset{x=1}{\sum }}{\left(\overset{g_{m}^{\left(i\right)}}{\underset{y=1}{\sum }}{\left(P_{m}^{\left(i\right)}\right)}_{xy}^{p}\right)}^{\frac{q}{p}}\right)}^{\frac{1}{q}}$$

Finally, the objective function of MLFLHMDA is mathematically formulated as follows:

(13) $$\begin{array}{l} \underset{G_{m},P_{m}^{\left(i\right)}}{\min}\overset{M}{\underset{i=1}{\sum }}{\left\|F_{m}^{\left(i\right)}{\left(P_{m}^{\left(i\right)}\right)}^{T}-G_{m}\right\|}_{F}^{2}+\alpha {\left\|G_{m}-A\right\|}_{F}^{2}\\ +tr\left(G_{m}^{T}\left(L_{m}\right)G_{m}\right)+\lambda \overset{M}{\underset{i=1}{\sum }}{\left\|P_{m}^{\left(i\right)}\right\|}_{1,2}^{2}s.t.P_{m}^{\left(i\right)}\geq 0 \end{array}$$

here λ is a regularization coefficient used to control the sparsity of $P_{m}^{\left(i\right)},i=1,2$ . The first term of the equation is the projection of different feature matrices into a common latent interaction subspace. The second term ensures that the predicted matrix $G_{m}$ approximates the known associated matrix A . The third term is a graph of Laplacian regulation. Finally, the two regularization coefficients α and λ are set to $10^{2}$ and $10^{-3}$ respectively as suggested by Xiao et al. (2020).

### Optimization

To solve the optimization problem in Eq. (13), we employ an iterative parameter method, alternately updating $G_{m}$ and $P_{m}$ to obtain the optimal solution, thus obtaining the corresponding prediction matrix.

Fix $P_{m}^{\left(i\right)}$ , and solve for $G_{m}$ . The optimization problem is reduced to the following sub-problem for $G_{m}$ :

(14) $$\underset{G_{m}}{\min}\overset{M}{\underset{i=1}{\sum }}{\left\|F_{m}^{\left(i\right)}{\left(P_{m}^{\left(i\right)}\right)}^{T}-G_{m}\right\|}_{F}^{2}+\alpha {\left\|G_{m}-A\right\|}_{F}^{2}+tr\left(G_{m}^{T}\left(L_{m}\right)G_{m}\right)$$

By differentiating Eq. (14) and setting it to zero, the update rule for $G_{m}$ can be derived as follows:

(15) $$G_{m}={\left(L_{m}+\left(M+\alpha \right)I\right)}^{-1}\left(\alpha A+\overset{M}{\underset{i=1}{\sum }}F_{m}^{\left(i\right)}{\left(P_{m}^{\left(i\right)}\right)}^{T}\right)$$

Fix $G_{m}$ , and solve for $P_{m}^{\left(i\right)}$ . Similarly, the optimization problem is reduced to the following sub-problem for $P_{m}^{\left(i\right)}$ :

(16) $$\begin{array}{l} \underset{P_{m}^{\left(i\right)}}{\min}\overset{M}{\underset{i=1}{\sum }}{\left\|F_{m}^{\left(i\right)}{\left(P_{m}^{\left(i\right)}\right)}^{T}-G_{m}\right\|}_{F}^{2}+\lambda \overset{M}{\underset{i=1}{\sum }}{\left\|P_{m}^{\left(i\right)}\right\|}_{1,2}^{2}\;\\ s.t.P_{m}^{\left(i\right)}\geq 0 \end{array}$$

To optimize Eq. (16), we introduce the Lagrange multiplier $\varphi^{\left(i\right)}$ for the constraint of $H_{m}^{\left(i\right)}$ , and thus the sub-problem is formulated as shown in Eq. (17):

(17) $$\begin{array}{l} f\left(P_{m}^{\left(i\right)},\varphi^{\left(i\right)}\right)=\overset{M}{\underset{i=1}{\sum }}{\left\|F_{m}^{\left(i\right)}{\left(P_{m}^{\left(i\right)}\right)}^{T}-G_{m}\right\|}_{F}^{2}+\lambda \overset{M}{\underset{i=1}{\sum }}{\left\|P_{m}^{\left(i\right)}\right\|}_{1,2}^{2}\\ -tr\left(\varphi^{\left(i\right)}\overset{M}{\underset{i}{\sum }}P_{m}^{\left(i\right)}\right) \end{array}$$

The partial derivative of the function


$$f\left(P_{m}^{\left(i\right)},\varphi^{\left(i\right)}\right)\mathrm{with respect to}P_{m}^{\left(i\right)}$$

is given by Eq. (18):

(18) $$\frac{\partial f\left(P_{m}^{\left(i\right)},\varphi^{\left(i\right)}\right)}{\partial P_{m}^{\left(i\right)}}=2P_{m}^{\left(i\right)}{\left(F_{m}^{\left(i\right)}\right)}^{T}F_{m}^{\left(i\right)}-2G_{m}^{T}F_{m}^{\left(i\right)}+2\lambda P_{m}^{\left(i\right)}ee^{T}-\varphi^{\left(i\right)}$$

Then, setting the derivative to zero and utilizing the KKT condition ${\left(\varphi^{\left(i\right)}\right)}_{jk}{\left(P_{m}^{\left(i\right)}\right)}_{jk}$ = 0, we have:

(19) $${\left(P_{m}^{\left(i\right)}\right)}_{jk}={\left(P_{m}^{\left(i\right)}\right)}_{jk}⊙\frac{{\left(G_{m}^{T}F_{m}^{\left(i\right)}\right)}_{jk}}{{\left(P_{m}^{\left(i\right)}\left({\left(F_{m}^{\left(i\right)}\right)}^{T}F_{m}^{\left(i\right)}+\lambda ee^{T}\right)\right)}_{jk}}$$

where ${\left(P_{m}^{\left(i\right)}\right)}_{jk}$ is defined as the element in the j - th row and k - th column of matrix $P_{m}^{\left(i\right)}$ , ⊙ denotes the Hadamard product in Eq. (19). $e\in {\left\{1\right\}}^{g_{m}^{\left(i\right)}\times 1}$ is a vector and $g_{m}^{\left(i\right)}$ denotes the number of features in the feature matrix $F_{m}^{\left(i\right)}$ .

Considering that elements in $P_{m}^{\left(i\right)}$ may be negative, the modified iterative equation for $P_{m}^{\left(i\right)}\;$ is as follows:

(20) $${\left(P_{m}^{\left(i\right)}\right)}_{jk}={\left(P_{m}^{\left(i\right)}\right)}_{jk}⊙\sqrt{\frac{{\left(P_{m}^{\left(i\right)}{\left({\left(F_{m}^{\left(i\right)}\right)}^{T}F_{m}^{\left(i\right)}+\lambda ee^{T}\right)}^{+}+{\left(G_{m}^{T}F_{m}^{\left(i\right)}\right)}^{-}\right)}_{ij}}{{\left(P_{m}^{\left(i\right)}{\left({\left(F_{m}^{\left(i\right)}\right)}^{T}F_{m}^{\left(i\right)}+\lambda ee^{T}\right)}^{-}+{\left(G_{m}^{T}F_{m}^{\left(i\right)}\right)}^{+}\right)}_{ij}}}$$

where the matrices with negative and positive symbols are defined as $X_{jk}^{-}=\left(\left|X_{jk}\right|-X_{jk}\right)/2$ and $X_{jk}^{+}=\left(\left|X_{jk}\right|+X_{jk}\right)/2$ .

Finally, we can utilize Eq. (15) and Eq. (20) to perform alternating iterations on $G_{m}$ and $P_{m}^{\left(i\right)}$ until convergence.

The above optimization is conducted from a microbe view, while the model and optimization from a disease view are similar. Table 2 describes the MLFLHMDA method to predict new microbe-disease associations. The original information’s adjacency matrix is represented as $A^{T}$ , SD represents disease similarity profiles, and $F={\left\{F_{d}^{\left(i\right)}\in R^{n\times g_{d}^{\left(i\right)}}\right\}}_{i}^{M}$ represents the disease feature matrix. Finally, the prediction matrices from the microbe and disease views are averaged and weighted to obtain the final matrix, denoted as matrix $G=\left(G_{m}+G_{d}\right)/2$ , the values of the entities in the matrix G stand for the pairwise correlation scores between microbes and diseases.

**Table 2:** Description of algorithm MLFLHMDA.

Algorithm MLFLHMDA
Input: microbe-disease association adjacency matrix A , microbe GIP similarity matrix SM , microbe feature matrices ${\{F_{m}^{\left(i\right)}\}}_{i=1}^{M}$ , disease GIP similarity matrix SD , disease matrices ${\{F_{d}^{\left(i\right)}\}}_{i=1}^{M}$ parameters α , λ Output: the predicted association matrix G Initialize ${\left\{P_{m}^{\left(i\right)}\in R^{n\times g_{m}^{\left(i\right)}}\right\}}_{i=1}^{M}$ and ${\left\{P_{d}^{\left(i\right)}\in R^{m\times g_{d}^{\left(i\right)}}\right\}}_{i=1}^{M}$ with random values in the interval [0,1] update the interaction profiles of each microbe $m_{i}$ and each disease $d_{i}$ with WKNKN by Eq. (5) and Eq. (6) $A_{md}=\left(\varsigma_{1}A_{m}+\varsigma_{2}A_{d}\right)/\sum \varsigma_{i}\left(i=1,2\right)$ update microbe-disease association adjacency matrix A by Eq. (8) Repeat: //From microbe view update $G_{m}$ by Eq. (15) with fixing ${\left\{P_{m}^{\left(i\right)}\right\}}_{i=1}^{M}$ for i←1 to M do update $P_{m}^{\left(i\right)}$ by Eq. (20) with fixing $G_{m}$ end for until convergence Repeat: //From disease view update $G_{d}$ and $P_{d}^{\left(i\right)}$ with the similar rules until convergence obtain the predicted association matrix G by $G=\left(G_{m}+G_{d}\right)/2$ return G

## Results

### Performance evaluation

To evaluate the predictive performance of the MLFLHMDA, we conduct global LOOCV and 5-fold cross validation using the HMDAD database with validated associations. In global LOOCV, each known association sample is treated sequentially as a test sample, with the remaining associations used as training samples, while unvalidated microbe-disease associations are considered candidate samples. In each round, the test sample is transformed into an unvalidated status and used to test the model. The test sample is ranked based on its predicted score among all candidate samples, and it is considered a correct prediction only when the test sample’s rank exceeds a certain threshold. In the 5-fold cross validation, akin to global LOOCV, we randomly divide the observed microbe-disease associations into five groups. Each group serves as a test sample in turn, while the remaining four groups are used as training samples for model learning. Unvalidated associations are considered candidate samples. We perform 100 times random partitioning in the 5-fold cross validation to mitigate potential errors arising from the random allocation of sample regions. After configuring a range of thresholds, we plot the Receiver operating characteristic (ROC) curve with the true positive rate (TPR, sensitivity) on the horizontal axis and the false positive rate (FPR, 1-specificity) on the vertical axis. Ultimately, we compute the Area Under the ROC Curve (AUC) as a fundamental performance evaluation metric.

**Figure 2:**
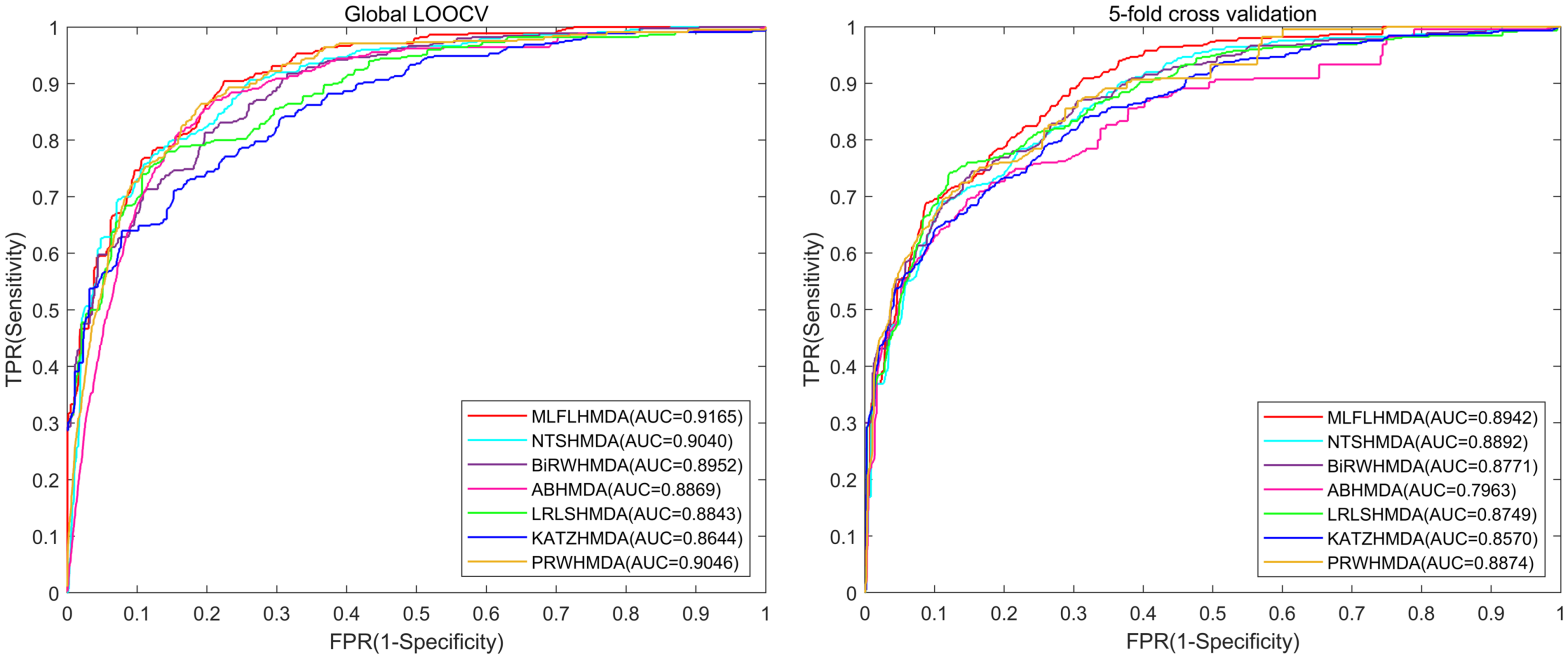
Performance comparison between MLFLHMDA and six classical microbe-disease association prediction models (NTSHMDA, BiRWHMDA, ABHMDA, LRLSHMDA, KATZHMDA, and PRWHMDA) in terms of ROC curves and AUCs based on global LOOCV and 5-fold cross validation. As a result, MLFLHMDA outperformed other models by achieving an AUC of 0.9165 in global LOOCV and an AUC of 0.8942 in 5-fold cross validation.

As shown in Figure 2, under global LOOCV, the AUC of MLFLHMDA is 0.9165, which is 1.25, 2.13, 2.96, 3.22, 5.21 and 1.19% higher than that of NTSHMDA (Luo and Long, 2020), BiRWHMDA (Zou et al., 2017), ABHMDA (Peng et al., 2018), LRLSHMDA (Wang et al., 2017), KATZHMDA (Chen et al., 2017), and PRWHMDA (Wu et al., 2018) respectively. Similarly, under 5-fold cross validation, the AUC of MLFLHMDA is 0.8942, which is 0.5, 1.71, 9.79, 1.93, 3.72, and 0.68% higher than that of NTSHMDA, BiRWHMDA, ABHMDA, LRLSHMDA, KATZHMDA and PRWHMDA, respectively.

### Ablation study

Ablation experiments are performed on the model under global leave-one-out cross-validation, and the effects of graph regularization and $L_{p,q}$ -norms are evaluated separately. Table 3 shows that both graph regularization and $L_{p,q}$ -norms improved the performance of the model. It is due to the ability of graph regularization to effectively capture the graph structure information of the data space and constrain the optimization iterations. The $L_{p,q}$ -norms also improve model performance by reducing noise in the data space and capturing the most characteristic features.

**Table 3:** Comparison of adding graph regularization and $L_{p,q}$ -norms in global leave-one-out cross-validation.

	MLFLHMDA	No graph regularization	No $L_{p,q}$ -norms	No graph regularization and $L_{p,q}$ -norms
AUC	0.9165	0.8919	0.8990	0.8898

### Case study

To further validate the predictive capabilities of the MLFLHMDA, we conduct two independent case studies involving significant human health conditions. In the first case study, we sort all unknown samples under the same disease and verify whether the association between the top 10 microbes and the disease under study is validated by the relevant literature. In the second case study, the aim is to assess the model’s capability to predict associations between unknown microbes and diseases in the absence of any known relevant microbe. Specifically, we reset all microbe associations for a particular disease in the adjacency matrix to zero. After model predictions, we verify the number of microbe samples within the top 10 rankings for diseases that are confirmed by relevant literature. In this context, we conduct the first case studies for asthma, colon cancer, and inflammatory bowel disease, and the second case study for Type 1 diabetes. The number of confirmed results from the literature for these four diseases is 10, 9, 9, and 8, respectively.

Asthma is a chronic disease that affects the airways of the lungs and is one of the most common respiratory disorders (Wu et al., 2019). According to statistics, asthma affects over 300 million people worldwide, and it is estimated to increase to 400 million people by the year 2025 (Barcik et al., 2020). Although the exact mechanisms underlying asthma remain unclear, the disease is associated with a variety of genetic, environmental, infectious, and nutritional factors. For instance, probiotics like Lactobacillus (First in the prediction list) can effectively treat allergic diseases or gastrointestinal inflammation. Research indicates that asthma patients have a disproportionately high proportion of Proteobacteria compared to non-asthmatic individuals, including certain pathogenic bacteria that may cause acute respiratory illnesses, such as Burkholderia species (Second in the prediction list) and Pseudomonas (Third in the prediction list) (Huang et al., 2011; Beigelman et al., 2014). In this study, there are 9 out of the top 10 microbes predicted by MLFLHMDA have been experimentally validated to be associated with asthma, as shown in Table 4.

**Table 4:** Prediction results for the top 10 asthma-associated microbes.

Rank	Microbe	Evidence
1	Lactobacillus	PMID:20592920
2	Burkholderia	PMID:24451910
3	Pseudomonas	PMID:9294308
4	*Clostridium difficile*	PMID:21872915
5	*Clostridium coccoides*	PMID:21477358
6	Actinobacteria	PMID:23265859
7	*Tropheryma whipplei*	PMID:26647445
8	Bifidobacterium	PMID:24735374
9	Firmicutes	PMID:23265859
10	Streptococcus	PMID:17950502

Colorectal cancer (CRC) ranks as one of the most prevalent cancer types, occupying the third position in terms of the incidence of malignant tumors. Furthermore, it stood as the second leading cause of cancer-related mortality in the year 2020 (Ou et al., 2023). Studies have indicated a close association between gut microbe and the onset of colon cancer (Marmol et al., 2017). For example, Proteobacteria (First in the prediction list) is a significant bacterial taxonomic unit, and in colorectal carcinoma tissues, there is a higher abundance of Corynebacterium (Zhu et al., 2014). In addition, a strong association of spontaneous *C. septicum* (Third in the prediction list) infection with hematological and colorectal malignancies has been reported (Larson et al., 1995; Ramkissoon et al., 2000; Kennedy et al., 2005; Powell et al., 2008). In this study, there are 9 out of the top 10 microbes predicted by the model have been experimentally validated to be associated with colon cancer, as shown in Table 5.

**Table 5:** Prediction results for the top 10 colorectal cancer-associated microbes.

Rank	Microbe	Evidence
1	Proteobacteria	PMID:24603888
2	*Tropheryma whipplei*	Unconfirmed
3	*Clostridium coccoides*	PMID:19807912
4	*Clostridium difficile*	PMID:19807912
5	Bifidobacterium	PMID:9111222
6	*Helicobacter pylori*	PMID:11774957
7	*Staphylococcus aureus*	PMID:7074582
8	Actinobacteria	PMID:26811603
9	Hemophilus	PMID:22761885
10	Streptococcus	PMID:32920015

Inflammatory Bowel Disease (IBD) is a group of chronic inflammatory gastrointestinal disorders, including Crohn’s disease and ulcerative colitis (Baumgart and Carding, 2007). While the exact causes of IBD are not fully understood, it is generally believed to involve genetic, environmental, and immune factors. There is already evidence suggesting a close connection between the gut microbe and IBD. For example, in IBD, especially in different variants, a reduction in Bacteroidetes (First in the prediction list) and Firmicutes (Eighth in the prediction list) occurs (Walters et al., 2014). Furthermore, studies propose that in IBD patients, especially during active phases, bacteria from the *Clostridium coccoides* group (Second in the prediction list), such as *Faecalibacterium prausnitzii*, as well as Firmicutes and Bifidobacteria, are less abundant in the gut microbe, this may be associated with disease onset and the protection of the intestinal mucosa (Sokol et al., 2009). In this study, there are 9 out of the top 10 microbes predicted by the model have been experimentally validated to be associated with Inflammatory Bowel Disease, as shown in Table 6.

**Table 6:** Prediction results for the top 10 inflammatory bowel disease-associated microbes.

Rank	Microbe	Evidence
1	Bacteroidetes	PMID:25307765
2	*Clostridium coccoides*	PMID:19235886
3	*Tropheryma whipplei*	Unconfirmed
4	*Clostridium difficile*	Azimirad et al. (2012)
5	*Prevotella copri*	PMID: 36644130
6	*Helicobacter pylori*	PMID:22221289
7	Prevotella	PMID:25307765
8	Firmicutes	PMID:25307765
9	*Oxalobacter formigenes*	PMID:15610315
10	*Staphylococcus aureus*	Azimirad et al. (2012)

Type 1 Diabetes mellitus (T1DM) is an autoimmune-mediated chronic disease, accounting for 5–10% of diabetes cases. It is characterized by the destruction of the pancreatic beta cells that produce insulin, driven by the autoimmune system (Liu et al., 2023). Studies have suggested that the gut microbe may play a role in regulating glucose levels, potentially impacting energy balance and nutrient absorption (Liu et al., 2023). Some researchers find that, in T1D patients, the analysis of next-generation sequencing (NGS) of the microbiota reveals an increased abundance of *Prevotella copri* (Third in the prediction list) at the time of disease onset (Traversi et al., 2022). In our second case study on Type 1 diabetes, we assess the predictive capability of MLFLHMDA for potential microbe-related diseases. The results reveal that among the top 10 predicted microbiota associated with the disease, eight show varying degrees of validation. Six of these associations are confirmed using the HMDAD database. The specific ranking is shown in Table 7.

**Table 7:** Prediction results for the top 10 type 1 diabetes mellitus-associated microbes.

Rank	Microbe	Evidence
1	*Tropheryma whipplei*	Unconfirmed
2	*Clostridium difficile*	Unconfirmed
3	*Prevotella copri*	PMID: 36562032
4	Clostridia	Confirmed
5	Proteobacteria	Confirmed
6	Lactobacillus	Confirmed
7	Bacteroides	Confirmed
8	Bacteroidetes	Confirmed
9	Prevotella	Confirmed
10	*Clostridium coccoides*	PMID: 23433344

The case studies on the four complex human diseases have confirmed the outstanding predictive capabilities of MLFLHMDA. To facilitate further research and validation, we provide the probability rankings for all unconfirmed disease-microbe associations (See Supplementary Table 1). It is expected that the highly ranked candidate microbe-disease pairs will offer valuable leads and will be experimentally verified shortly.

## Conclusion

Microbes play a significant role in human diseases and physiological processes. They are numerous, diverse, and interconnected within ecosystems. Exploring the potential associations between microbes and diseases is beneficial for maintaining human health and understanding disease mechanisms.

In this study, we develop a prediction model called MLFLHMDA based on multi-view latent feature learning. This method infers microbes associated with diseases from both microbe and disease views. Since the lack of known microbe-disease associations leads to sparsity in the microbe-disease association matrix, we preprocess the association matrix using WKNKN to add more interaction information. Additionally, we construct Gaussian interaction profile kernel similarity between microbes and diseases and use PCA to extract potential features in GIP kernel similarity. To obtain more potential information, multi-modal potential features are projected into the common subspace. We impose graph regularization and $L_{p,q}$ -norms in the integrated latent feature learning to enhance the model’s generalization performance, and score and rank each microbe and disease pair in the alternating iteration algorithm. In global LOOCV and 5-fold cross validation, the AUC of MLFLHMDA is 0.9165 and 0.8942, respectively, which outperforms the other six methods. What’s more, case studies of four diseases further validate the good predictive performance of the model.

However, MLFLHMDA still has certain limitations that need to be addressed in future work. The model solely relies on the HMDAD dataset, and collecting more experimentally validated disease-microbe relationships could improve the predictive ability of MLFLHMDA. In future work, we intend to integrate more association information as well as similarity information, and combine the advantages of existing models to construct a more superior predictive model. We believe that this method could guide medical experiments to get the potential associations and inspire in other bioinformatics fields such as circRNA-disease association prediction (Wang et al., 2021), MiRNA-disease association prediction (Chen et al., 2018), and so on. In addition, considering the high application value of genetic information, the introduction of the genetics information of the host in the future microbe-disease prediction field will inevitably benefit the association prediction. What is more, after more and more potential microbe-disease associations are predicted and confirmed, we could further predict the association between microbes and drugs based on the microbe-disease association information and other related data, which is favorable to provide new strategy design for drugs and human disease treatment (Han et al., 2023).

## Data availability statement

The original contributions presented in the study are included in the article/Supplementary material, further inquiries can be directed to the corresponding author.

## Author contributions

ZC: Conceptualization, Supervision, Writing – review & editing. LZ: Data curation, Formal analysis, Investigation, Methodology, Software, Validation, Writing – original draft. JL: Formal analysis, Software, Validation, Writing – original draft. MF: Data curation, Investigation, Validation, Writing – original draft.

## Footnotes

^1^ http://www.cuilab.cn/hmdad
