# Supplementary material for: MLFLHMDA: predicting human microbe-disease association based on multi-view latent feature learning
Source: Front Microbiol. 2024 Feb 2;15:1353278. doi: 10.3389/fmicb.2024.1353278 (PMC10869561; doi:10.3389/fmicb.2024.1353278)
Supplement: Supplementary file 1 [file Data_Sheet_1.ZIP › Supplementary_Material/Table 1.DOCX]

Supplementary Material

MLFLHMDA: Predicting Human Microbe-Disease Association Based on Multi-view Latent Feature Learning

Ziwei Chen^#, *^, Liangzhe Zhang^#^, Jingyi Li, Mingyang Fu

School of Electronic and Information Engineering, Beijing Jiaotong University, Beijing, 100044, China

*** Correspondence:**Ziwei Chen
[zwchen@bjtu.edu.cn](mailto:zwchen@bjtu.edu.cn)

**#Joint First Authors:**

Ziwei Chen, Liangzhe Zhang

Keywords: microbe, disease, microbe-disease association, multi-view, latent feature learning

# Supplementary Table

**Supplementary Table 1.** We applied MLFLHMDA to prioritize all the candidate microbe-disease pairs based on all the known microbe-disease associations in HMDAD database as training samples. This prediction result is released for further experimental validation and research.
